# Mr. Jones, on Nitrat of Silver in Hooping Cough

**Published:** 1806-07

**Authors:** Edward Jones

**Affiliations:** Member of the College of Surgeons. Hall, Montgomeryshire


### *To the Editors of the Medical and Phyſical Journal.*

GENTLEMEN,

HAVING experienced much vexation and disappointment in the employment of all the usual remedies for hooping cough, I was induced, by the very powerful effects of the nitrat of silver in diseases somewhat analogous, to make trial of it; the first instances in which I used it justified a perseverance, and I can now, from pretty extensive experience, assert, that it possesses at least *equal* efficacy to any other medicine hitherto recommended in that troublesome disorder; I have always given it in the form of pill, made up with the smallest possible quantity of bread crumb, in which state, from the very minute size of the pills, it possesses the advantage of being easily concealed in a little jelly, or any substance of a similar nature, which a child can be prevailed upon to take.

I am, &c.

*Hall, Montgomeryshire,*
*May* 24, 1106.

EDWARD JONES,
Member of the College of Surgeons.

